# Reconfigurable Laser-Stimulated Lock-In Thermography for Surface Micro-Crack Detection

**DOI:** 10.3390/s23084090

**Published:** 2023-04-19

**Authors:** Lu Ding, Sergey Gorelik, Pei Wang, Anton Valentinovich Sadovoy, Qiang Zhu, Andrew Chun Yong Ngo, Jinghua Teng

**Affiliations:** 1 Institute of Materials Research and Engineering (IMRE), Agency for Science, Technology and Research (A*STAR), 2 Fusionopolis Way, Innovis #08-03, Singapore 138634, Singapore; 2 Singapore Institute of Food and Biotechnology Innovation, Agency for Science, Technology and Research (A*STAR), Singapore 138669, Singapore

**Keywords:** lock-in thermography, reconfigurable spatial light modulator, surface micro-crack detection, non-destructive evaluation, infrared thermography

## Abstract

Surface crack detection and sizing is essential for the manufacturing and maintenance of engines, run parts, and other metal elements of aircrafts. Among various non-destructive detection methods, the fully non-contact and non-intrusive technique based on laser-stimulated lock-in thermography (LLT) has recently attracted a lot of attention from the aerospace industry. We propose and demonstrate a system of reconfigurable LLT for three-dimensional surface crack detection in metal alloys. For large area inspection, the multi-spot LLT can speed up the inspection time by a factor of the number of spots. The minimum resolved size of micro-holes is ~50 µm in diameter limited by the magnification of the camera lens. We also study the crack length ranging from 0.8 to 3.4 mm by varying the modulation frequency of LLT. An empirical parameter related to the thermal diffusion length is found to show the linear dependence with the crack length. With the proper calibration, this parameter can be used to predict the sizing of the surface fatigue cracks. Reconfigurable LLT allows us to quickly locate the crack position and accurately measure its dimensions. This method is also applicable to the non-destructive detection of surface or sub-surface defect in other materials used in various industries.

## 1. Introduction

In the manufacturing and maintenance of machinery components and critical structures in transportation and other industries, it is crucial to rapidly and accurately detect and size surface cracks [1] in a variety of materials, including metals, ceramics, and composites. Various detection methods exist, such as dye penetrant, Eddy current, magnetic particle, ultrasonic, and visual inspection. Dye penetrant testing [2,3] applies a liquid dye to the surface of a material, which penetrates any surface-breaking defects, such as cracks or porosity. This method is low cost but invasive, which poses a risk of surface contamination and requires tedious post-cleaning. Eddy current testing [4] based on electromagnetic induction and magnetic particle testing [5] based on magnetic flux leakage only apply to conductive and ferromagnetic materials, respectively. Ultrasonic inspection [6] is a method of characterizing the surface or internal defects of a test piece using high frequency sound waves. It requires direct contact between the ultrasound transducer and the testing materials, making it unsuitable for non-contact inspection. The light scattering techniques [7] such as scatterometry can infer surface information from scattering patterns. This non-invasive and fast visual inspection method is widely used in material analysis and small particle sizing. The strength of these techniques lies in the robust theories that exist to analyze and interpret the data. However, it’s important to understand that these theories rely on certain key assumptions. Without proper consideration of these assumptions in the context of the measurement and material being analyzed, the quality of the results may be questionable. One issue that can lead to false-positive alarms is the presence of surface contaminations or scratches, which can significantly impact the accuracy of the measurements. Additionally, changes in illumination conditions can also impact the results, making it important to carefully control the experimental setup to ensure reliable data. Therefore, a fully non-contact, non-destructive and non-intrusive technique which can be applied to a wide range of materials is mostly preferred.

**Figure 1:**
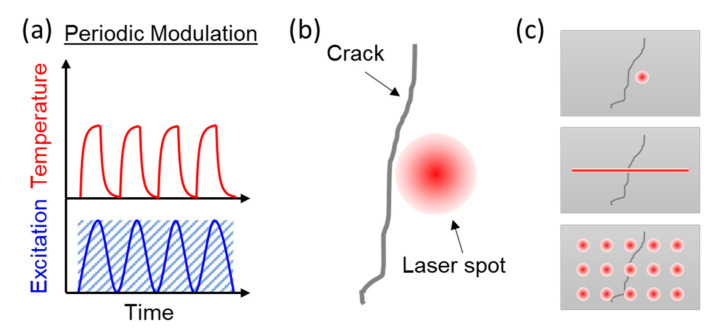
(**a**) Time domain optical excitation and surface temperature profile at the excitation point under periodic modulation. (**b**) The schematic of an open crack with a laser spot in its vicinity. The diameter of the laser spot is chosen to confine 1/e^2^ of the intensity. (**c**) Surface crack detection schemes using a single spot, a single line, and multi-spots by a predesigned diffractive optical element.

Optical-stimulated lock-in infrared thermography [1,8,9,10] is gaining popularity as a non-contact, non-destructive and non-intrusive technique for crack detection due to good signal-to-noise ratio and high accuracy. It relies on the principles of infrared thermography (typically between 3 and 14 μm), which involves the use of infrared radiation to detect and measure temperature variations in a material. The technique works by applying a modulated light source to heat up the surface of the material being inspected. The light sources can be a lamp or laser corresponding to an extended or localized optical radiation, respectively. The surface is heated up due to material absorption and the heat is dissipated into the solid via conduction. Any discontinuity of the thermal diffusivity, i.e., an interface, encountered by the thermal wave along its propagation direction will cause reflection, refraction, and scattering and result in a sudden change in the amplitude and phase of the thermal wave. Therefore, localized heat source such as a tightly focused laser spot generates lateral propagating thermal wave and is particularly suitable to detect surface cracks. In addition, the light intensity in lock-in thermography is sinusoidally modulated to generate periodic temperature-time profile on the surface. A simple diagram is shown in Figure 1a. The modulation frequency as a reference signal mixes with the infrared radiation signal modulated at the same frequency and synchronized with the infrared camera. The lock-in signal undergoes narrow band filtering and DC background removal which gives rise to the high signal-to-noise ratio and makes the technique insensitive to non-uniform heating, local variations in surface emissivity, surface irregularities, and external reflections. By using the lock-in principle, this technique is able to extract weak signals from a noisy background, making it highly effective for crack detection even in challenging environments. In addition, compared with other infrared thermography techniques, optical-stimulated lock-in infrared thermography requires fewer post-image processing methods to enhance the detection accuracy and quantification of material damages. With its high accuracy and signal-to-noise ratio, optical-stimulated lock-in infrared thermography provides an efficient and effective means of detecting surface cracks and other types of material damage.

The laser-stimulated lock-in thermography (LLT) technique [11,12,13] that utilized a single-spot laser source for heat generation and an infrared camera for crack inspection is demonstrated. As shown in Figure 1b, the focused laser spot is positioned at the vicinity of the middle of the crack for accurate size detection. Heat wave propagates from the center of the spot to the surroundings. Thermal gradient in the direction perpendicular to the crack is built up. The crack causes the discontinuity of the heat distribution and so the position of the crack is revealed. The larger the thermal discontinuity, the better the accuracy of the crack detection. The actual size and position of the laser spot need to be carefully adjusted according to the material’s thermal property. Typically, the precision of positioning needs to be controlled in sub-mm scale compared to the size of the laser spot (approximately 1~2 mm). The actual distance depends on the thermal diffusion length of the thermal wave propagating in the material. If the laser is positioned too far away from the crack, the thermal discontinuity is buried in the noise floor. The crack also becomes invisible when the laser spot sits on the crack. Surface crack detection schemes using a single spot, a single line, and multi-spots created by a predesigned diffractive optical element (DOE) are also reported, shown in Figure 1c. From a practical point of view, the single-spot LLT technique is less favorable for real-time inspection of large surface area due to its long scanning time and signal processing time to cover a large area. Single-line LLT [14] can greatly reduce the scanning time in one dimension, but risks covering the crack locations and thus missing the cracks. To avoid this, the number of scanning steps increase and eventually the time taken is similar to that of the single-spot method. A multi-spot LLT system was recently demonstrated for crack inspection of semiconductor chips during in-line manufacturing [15]. Multi-spots are generated by a DOE which is specially designed according to the material properties of the individual target. Since the DOE is a passive optical component which requires customized design to adapt to different excitation wavelengths and sample dimensions, it greatly hinders the flexibility and adaptability of such a LLT system.

Recently, we proposed a system of reconfigurable LLT for surface crack detection [16]. A spatial light modulator (SLM) is applied in the LLT system to modify the wavefront of incident laser beam and generates various beam patterns projecting on the sample. Compared to the existing LLT methods, the illumination beam pattern can be programmed and dynamically controlled by the spatial light modulator. The adjustment is very flexible and adaptive based on the sample conditions. Arranging the illumination pattern properly, our method can greatly reduce the overall scanning time, save the need for repeated optical alignment when changing samples and optics, while keeping the advantages of conventional LLT with low error rate, good signal-to-noise ratio and provide three-dimensional crack information on each individual crack. This method can also be applied to non-destructive detection of surface, sub-surface defects, or material boundary in semiconductors, composite materials, and many other materials. Here, to complete this study, we provide a better understanding of the reconfigurable LLT technique, regarding the modulation frequency, sample study, etc.

## 2. Experiment Description

**Figure 2:**
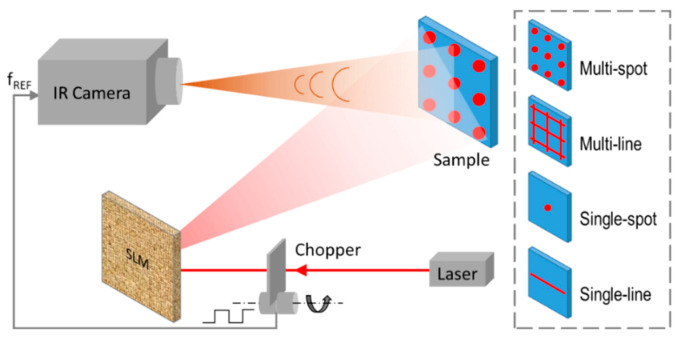
The schematic of a reconfigurable laser lock-in thermography system.

The reconfigurable LLT system is different from a conventional LLT system with an added active DOE element in the laser beam path. Figure 2 presents the schematic of the reconfigurable LLT system for surface crack detection. A laser beam is deflected by a spatial light modulator [17] (SLM, HOLOEYE Photonics AG) and then projected and focused on the sample. A spatial light modulator (SLM) is a device that can manipulate and control light by modifying its spatial profile. It is a type of optical modulator that can change the phase, amplitude, or polarization of light waves on a pixel-by-pixel basis, enabling the creation of complex optical patterns. Note that SLM is an electrically tuned DOE that shapes the wavefront of the incident laser beam. By configuring the SLM, the profile of the deflected laser beam can be switched between the following four configurations (not limited): (1) multi-spot, (2) multi-line, (3) single spot, and (4) single line. The spot size or line width and divergence angle for multi-spot or multi-line configurations are tunable in real time with a response time limited by SLM in milliseconds time scale. The laser beam is chopped for lock-in detection. The infrared camera (FLIR SC7500-BB) has a spectral range of 3–5 μm, a spatial resolution of 256 × 320 pixels and a thermal resolution of noise equivalent temperature difference (NETD) of <20 mK. The chopping frequency is sent to the infrared camera as a reference signal. The infrared CCD signals and the frequency reference signal are sent to the built-in lock-in detection module. The DC image as well as the amplitude and phase lock-in images are generated. The acquisition duration is a few cycles of the lock-in period.

This detection scheme is used to characterize microcracks in two types of metallic samples. Sample A is a porous nickel superalloy metal plate. It has many surface features and requires a large area inspection. For this sample, a multi-spot illumination configuration is applied to compare with the single-spot configuration. In the single-spot configuration, low modulation frequency/long data acquisition time are required to cover a large inspection area. With the multi-spot configuration, total inspection time can be saved. Sample B is a set of Ti-6Al-4V (Ti64) alloy metal blocks with thermal fatigue surface cracks (TrueFlaw, Espoo, Finland). Provided with the crack specifications, the single-spot LLT is used to analyze the crack sizing in detail and the data are fitted by an empirical formular. From the fitting, an empirical parameter is proposed for the calibration of the crack sizing.

## 3. Results and Discussions

### 3.1. Sample A: Porous Metal Sample

**Figure 3:**
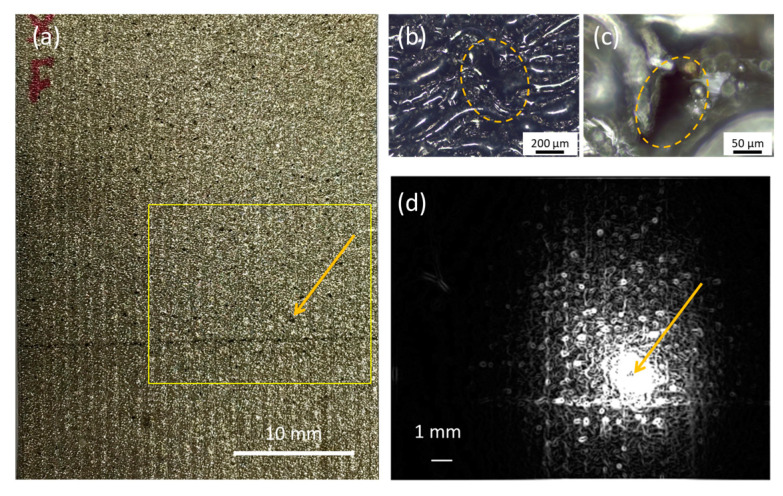
(**a**) A photo of a porous metal sample A with pores and cracks. The LLT inspection area is indicated as a yellow rectangle. An arrow indicates the location of a hole. (**b**,**c**) Zoom-in microscope images of the hole. (**d**) An amplitude image of single-spot LLT measurement at f = 0.0248 Hz.

Sample A is a piece of nickel superalloy metal block of 25 mm × 50 mm × 5 mm in dimension. It was fabricated using a metal additive manufacturing method, laser powder bed fusion [18]. Figure 3a shows a photo of a metal block covered with micro-pores and cracks. Figure 3b,c show zoom-in microscope images of typical micro-pores in details (dotted yellow circle). Typically, diameters of the micro-pores vary from 10 to 100 µm.

**Figure 4:**
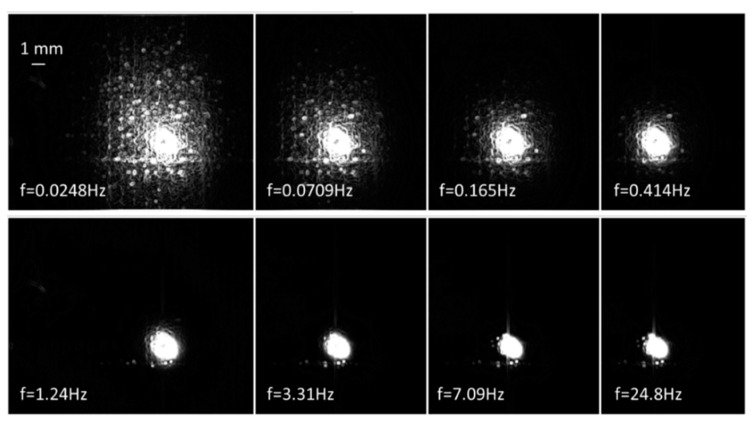
LLT amplitude images of sample A at different modulation frequency. Thermal diffusion length increases with decreasing modulation frequency, so that a larger area is covered. Total field of view is 15 × 18 mm^2^.

Figure 3d is the amplitude image taken by the single-spot LLT at a modulation frequency f = 0.0248 Hz. Temperature gradient has been established in an area of 10 mm^2^ with a center bright spot indicating the laser position. The image reveals many pores and cracks. Typically, 5–10 periods of excitation (200~400 s) are recorded for better signal-to-noise ratio. The size of the inspection area is related to the modulation frequency. Figure 4 shows LLT amplitude images of sample A at various modulation frequencies. At a high modulation frequency, only a bright laser spot is captured. While at low frequency, rich features of surface defects appears in a much larger area. It is because that the thermal diffusion length is inversely proportional to the square root of the modulation frequency [19]. Longer thermal diffusion length means larger temperature gradient covering area. However, longer data acquisition time is a trade-off. Therefore, single-spot detection has a trade-off between inspection area and data acquisition time.

**Figure 5:**
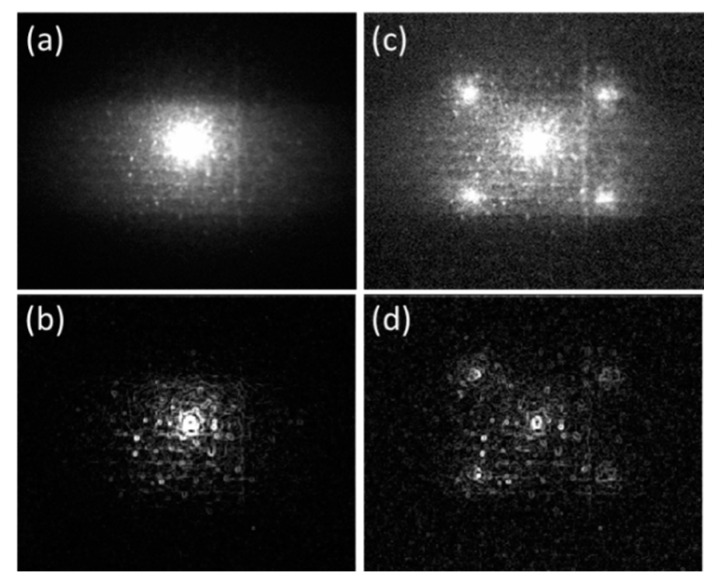
(**a**,**c**) are amplitude image of single-spot and multi-spot LLT measurement with f = 0.1 Hz. (**b**,**d**) are post-processed (**a**,**c**) by Sobel kernel, respectively.

Multi-spot illumination is a solution to the above-mentioned problem. With more illumination spots, the shorter thermal diffusion length is needed so the shorter data acquisition time. Figure 5 compares the amplitude images of single-spot and multi-spot LLT measurements at f = 0.1 Hz. Five spots, the zeroth diffraction order and ±1 diffraction order spots along two orthogonal directions, are generated by SLM and incident on the sample. The spot spacing depends on the actual thermal diffusion length which relates to the material thermal diffusivity and modulation frequency. It can be adjusted by the SLM setting as well as the distance between SLM and sample. Figure 5b,d are post-processed from Figure 5a,c, respectively, by Sobel kernel image processing [20]. Compared to single-spot measurement, multi-spot LLT at same modulation frequency covers a larger area and captures more surface defects. The minimum resolved size of micro-holes is ~50 µm in diameter limited by the magnification of the camera lens. In principle, an N × N array of laser spots can shorten the inspection time by a factor of N^2^. Total laser power is distributed into the N^2^ points for local heat generation. 5 points are chosen here as proof of concept because the limited total power of our laser. Here, no realignment is required to change the illumination configuration. The reconfigurable multi-spot/line scheme can obtain a large area overview of crack distribution with a minimum scanning effort, also can save time from repeated optical alignment when changing samples and the DOEs.

### 3.2. Sample B: Customized Fatigue Cracks in Ti64 Alloy

The single-spot/line scheme is more suitable for obtaining detailed three-dimensional information on a surface open crack on precise length, width, and depth with arbitrary orientation [12]. The acquisition settings depend on the optical and thermal properties of the target material. Here, we investigate customized surface fatigue cracks in Ti64 alloy (TrueFlaw, Finland). Ti64 alloy samples are square plates with size of 20 × 20 × 4 mm^3^ (Length × Width × Thickness). Table 1 lists the crack specifications, with cracks having a length from 0.8 mm to 3.4 mm and opening from 0.9 μm to 10 μm. With a given crack specification, the single-spot LLT is used to study the crack sizing.

**Figure 6:**
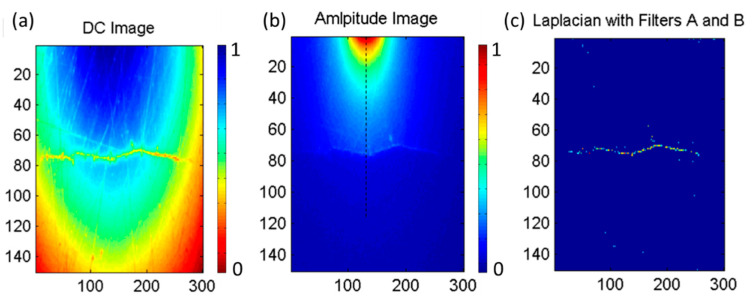
(**a**) DC image of a crack and surface scratches. Color map indicates the normalized relative temperature. (**b**) Lock-in amplitude image of the crack only with heating source. Color map indicates the normalized amplitude. Dashed line cuts the crack where the Laplacian is taken for crack sizing analysis in Figure 7b. (**c**) Processed lock-in amplitude image of the crack.

Figure 6 shows the experimental result on single-spot LLT detection of an open crack with irregular shape (length 1.7 mm and opening 2.0 um). The intensity-modulated laser stimulation is positioned at the vicinity of the crack. The temporal information (specifically, in the frequency domain) is precisely extracted by the lock-in detection method. The propagating thermal wave is described by the equation of conductive heat flow given by $\partial T/\partial t-\alpha \nabla^{2}T=0$, with α the thermal diffusivity and $\nabla^{2}T$ the Laplacian (the second-order spatial derivative) of the temperature. As these signals are often relatively weak compared to the background excitation, proper signal processing plays a crucial role in detecting signals associated with the presence of cracks. The 2D Laplacian of the amplitude image $\nabla^{2}T\left(x,y\right)$ can be used to highlight the useful signal, i.e., small crack-induced perturbations over a relatively smooth background. A three-step algorithm for the processing of the amplitude image is proposed to extract the surface defect [13]. Laplacian of the amplitude image reveals the discontinuity of thermal wave due to perturbation of cracks. Since the Laplacian of the amplitude is extremely sensitive to the heat source, amplitude threshold filters A (typically 80% of the maximum amplitude) is introduced to create masks to remove the heat source impact zone. It is also important to notice that the Laplacian of the signal in the vicinity of the crack is negative. Amplitude threshold filters B is applied to define the noise ceiling of the image. It is to clear any unwanted signal due to surface scratches. In the end, a binarization is performed to obtain the final image. Figure 6a shows the DC image in which not only the crack but also surface scratches are captured. It is difficult to identify the crack at this stage. In the lock-in amplitude image (Figure 6b), the crack blocks the thermal diffusion of the heat wave and results in the inhomogeneous thermal diffusion. Applying filters A and B, the crack is then clearly shown in a processed image in Figure 6c.

**Figure 7:**
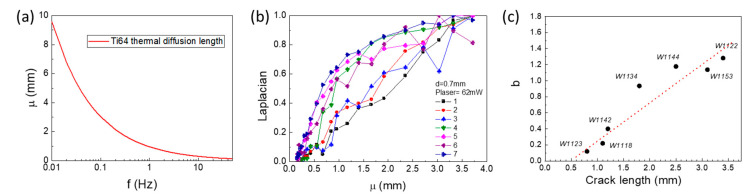
(**a**) Calculated thermal diffusion length μ as a function of modulation frequency f. For the sample cracks listed in Table 1, (**b**) normalized Laplacian of a crack as a function of thermal diffusion length μ for various cracks. Lock-in measurement taken at d = 0.7 mm and laser power 62 mW. (**c**) Correlation of parameter b to the crack length.

We then measure and analyze all samples listed in Table 1 based on the above given analysis algorithm. All cracks are successfully located in the thermal image. The heat source is located at a distance d to the crack along the perpendicular direction. The optimum d is chosen to be able to capture the crack within the thermal diffusion length μ. The thermal diffusion length μ is a function of modulation frequency f as μ = √(α/πf) = √((k⁄ρc_p_)/πf), where parameters are found in Table 2. Figure 7a plots the thermal diffusion length of Ti64 as a function of modulation frequency. For μ smaller than the distance d between the laser spot and the crack, the heat wave decays away before reaching the crack. In the special case where μ is comparable to d, the heat wave reaches the crack and is blocked. For longer diffusion lengths, the heat wave diffuses much further into the bulk along the crack as well as bypassing it. The temperature contrast is less significant. The choice of modulation frequency needs to cover this range as well as considering the laser power and camera sensitivity.

Figure 7b records the second-order spatial derivative (Laplacian) at the crack position as a function of thermal diffusion length. With frequency assisted imaging, we can distinguish crack with large length (W1122) from that with small length (W1123). We fit each curve with a simple and purely empirical exponential function $F\left(\mu \right)=A\left(1-e^{-b\mu }\right)$, where *A* is a scaling factor and *b* is in the unit of inverse of the length. Empirically, *b* indicates the approaching rate of the normalized Laplacian to 1 as a function of the diffusion length. Laplacian (the second-order spatial derivative) of the amplitude image reveals the discontinuity of thermal wave due to perturbation of cracks. It links to the capability of the thermal wave bypasses the crack. Laplacian is small for short crack #1, while it is large for large crack #7. Varying the diffusion length, normalized Laplacian approaches 1 faster in a large crack. So, *b* correlates well with the crack size which can be used as an indicator to calibrate the size. The correlation between parameter *b* and the crack length is shown in Figure 7c. The red dashed line is a guide to the eyes. Parameter *b* has linear dependence with the crack length and hence it should correlate to the crack depth as for fatigue cracks the crack depth is proportional to the crack length. Therefore, *b* can be used for predicting the crack depth with proper calibration. Such calibration using direct experimental measurements via other crack opening methods is required to tell the exact value of crack depth [19,21]. Further study is out of the scope of this work.

## 4. Conclusions

We demonstrate surface crack detection using a reconfigurable LLT. The SLM is used to generate various illumination configurations applied for different purposes. The multi-spot LLT, with five spots as demonstrated, can inspect an area of approximately 10 × 10 mm^2^ in 60 s, while the time for a single spot is 5-fold longer to cover the same area. Once it locates cracks, the single-spot LLT will be switched on for studying the individual cracks. Switching between different configurations can be performed by SLM and no realignment is required. We also investigate the size of fatigue cracks in Ti64 alloys by various modulation frequency LLT. An empirical parameter b relating to the thermal diffusion length shows linear dependence with the crack size. The tunability of the SLM enables the system to adapt to any material under investigation. It allows us to quickly locate the crack and accurately measure its dimension. Our method greatly reduces the overall scanning time, saves time from repeated optical alignment when changing samples and the DOEs, reduces the error rate, increases the signal-to-noise ratio and provides three-dimensional crack information on an individual crack. This method is also applicable to non-destructive detection of surface or hidden defect in semiconductors and integrated circuits. Lock-in thermography is a thermal-wave-based non-destructive testing technique. It may help to reveal the chemical composition or mechanical properties of the alloys if they cause changes in the material’s the thermal properties. The larger the thermal diffusion, the clearer the feature in lock-in thermography. Therefore, lock-in thermography is also used to detect hidden defects, such as void, delamination, and water ingress. Therefore, different heating methods, i.e., choices of wavelength, broadband or narrow-band excitation, need to be applied based on the nature of the defects.

## Figures and Tables

**Table 1:** List of the cracks in Ti64 alloy, including coordinates (X, Y), length and opening information.

No.	Crack ID	X/mm	Y/mm	Length/mm	Opening/μm
1	W1123	10	10	0.8	1.2
2	W1118	8	10	1.1	0.9
3	W1142	10	12	1.2	1.7
4	W1134	10	10	1.8	3.6
5	W1144	9	9	2.5	7
6	W1153	9	12	3.1	7
7	W1122	10	10	3.4	10

**Table 2:** Physical parameters of Ti64 Alloy.

Physical Parameters	Ti64 Alloy	Unit
Thermal conductivity k	6.7	Wm^−1^ K^−1^
Specific heat capacity c_p_	526.3	JKg ^−1^ K^−1^
Density ρ	4430	Kgm^−3^
Thermal diffusivity α = k/ρc_p_	2.87 × 10^−6^ @ 27 °C	m^2^ s^−1^

## Data Availability

Not applicable.
